# Bipolarity as an Independent Correlate of Clinical Profiles in Insomnia: A Factorial Analysis Across Multiple Clinical Domains

**DOI:** 10.1002/brb3.71642

**Published:** 2026-07-28

**Authors:** Sujin Kim, Seung Pil Pack, Heon‐Jeong Lee, Chul‐Hyun Cho

**Affiliations:** ^1^ Department of Psychiatry Korea University College of Medicine Seoul Republic of Korea; ^2^ Department of Biotechnology and Bioinformatics Korea University Sejong Republic of Korea; ^3^ Department of Biomedical Informatics Korea University College of Medicine Seoul Republic of Korea; ^4^ Department of Medical Education Korea University College of Medicine Seoul Republic of Korea

**Keywords:** anxiety, bipolarity, circadian rhythm, deep phenotyping, insomnia, mood disorder questionnaire

## Abstract

**Background:**

This study investigated the independent and interactive effects of bipolarity (subthreshold bipolar spectrum features) on multidimensional clinical profiles in individuals with insomnia, using data from a comprehensive deep phenotyping study.

**Methods:**

A total of 338 participants (252 with insomnia, 86 normal sleeper controls) from the SOMDAY deep phenotyping study were classified into a 2 × 2 factorial design: Sleep Status (Insomnia vs. Control) × Bipolarity (Korean version of the Mood Disorder Questionnaire [K‐MDQ] ≥ 7 vs. < 7). Two‐way analyses of covariance, adjusted for age and sex, were conducted across 14 validated clinical scales. Benjamini–Hochberg false discovery rate correction was applied, with rank‐transformed sensitivity analyses.

**Results:**

Sleep Status showed significant main effects across 13 of 14 scales (all false discovery rate‐corrected *p* < 0.05), with the largest effects on sleep quality (Pittsburgh Sleep Quality Index [PSQI], partial η2 = 0.230) and circadian rhythm disturbance (Korean version of the Biological Rhythms Interview of Assessment in Neuropsychiatry [K‐BRIAN], partial η2 = 0.210). Bipolarity was independently associated with elevations in 10 of 14 scales, with the strongest effects on anxiety (Generalized Anxiety Disorder‐7, partial η2 = 0.059), anxiety sensitivity (Body Sensations Questionnaire, partial η2 = 0.049), and depression (Patient Health Questionnaire‐9, partial η2 = 0.041). No interaction effects survived correction, indicating predominantly additive effects.

**Conclusion:**

Bipolarity, indexed by the K‐MDQ, exerts statistically independent main effects associated with greater psychological distress, anxiety sensitivity, and circadian vulnerability, operating additively with insomnia status across both insomnia patients and normal sleepers. These findings support the integration of bipolar spectrum screening into sleep medicine assessment.

## Graphical abstract

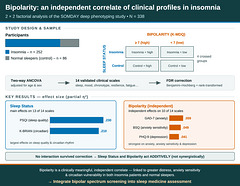

## 1 Introduction

Insomnia is one of the most prevalent sleep disorders, affecting approximately 10% of adults chronically and 30%–35% experiencing occasional symptoms (Morin and Buysse 2024; Morin and Jarrin 2022). It is characterized by difficulty in initiating or maintaining sleep despite adequate opportunity and is frequently accompanied by significant daytime impairment (Roth 2007). The etiology of insomnia involves multiple interacting factors, including genetic predisposition, psychological stress, maladaptive sleep behaviors, cognitive factors, circadian rhythm disruption, and comorbid psychiatric conditions (Lee et al. 2025; Morin and Jarrin 2022).

Conventional clinical assessment of insomnia has primarily relied on unidimensional measures such as sleep questionnaires and clinical interviews. Although polysomnography and actigraphy offer more objective assessment, their application is often limited to brief observation periods (Lee et al. 2025). A “deep phenotyping” approach has emerged as an alternative, integrating standardized clinical questionnaires, continuous digital phenotyping through wearable devices and smartphone applications, functional neuroimaging, and biomarker analyses within a comprehensive framework (Lee et al. 2025). The present study focuses on the clinical questionnaire component of this multimodal protocol.

Among factors that may shape the clinical presentation of insomnia, bipolar spectrum features—commonly referred to as “bipolarity”—remain underexplored in insomnia populations. Bipolarity refers to subthreshold manic or hypomanic symptoms existing along a continuum from normal mood variability to full bipolar disorder (Angst et al. 2012). Even below the diagnostic threshold, bipolar spectrum features are associated with disrupted circadian rhythms, heightened emotional reactivity, and vulnerability to sleep disturbances (Giglio et al. 2010; Harvey 2008). The Korean version of the Mood Disorder Questionnaire (K‐MDQ) enables identification of individuals with elevated bipolarity risk in clinical populations (Jon et al. 2009).

Bipolarity and insomnia share a bidirectional relationship with direct clinical relevance. Sleep disturbance is a core feature of bipolar spectrum conditions, and insomnia has been identified as both a prodromal marker and a precipitant of mood episodes (Harvey 2008, 2011). Individuals with elevated bipolarity scores tend to exhibit greater circadian rhythm disturbance and psychological distress (Cho et al. 2018; Giglio et al. 2010). However, most studies have examined bipolarity within diagnosed bipolar disorder, and its independent contribution to the clinical phenotype of insomnia has not been systematically investigated.

The present study therefore examined bipolarity effects across a broad set of clinical domains selected based on prior literature and clinical relevance. We included sleep‐related measures (PSQI, ESS, DBAS‐16, BPS, FIRST) as the primary clinical domain. Mood and anxiety assessments (PHQ‐9, GAD‐7, BSQ) were selected because affective dysregulation is a hallmark of bipolar spectrum conditions (Angst et al. 2012; Harvey 2008). Resilience (KRQ‐53) was included as a potential protective factor. Circadian rhythm measures (CSM, K‐BRIAN) were chosen given the well‐established link between circadian disruption and bipolar spectrum features (Cho et al. 2018; Giglio et al. 2010). Fatigue (MFS) was assessed as a common functional consequence of both conditions. Alcohol use (AUDIT‐K) was included as a known comorbidity in sleep and mood disorder populations. Finally, seasonal mood variation (SPAQ GSS) was assessed given the established connection between bipolar spectrum conditions and seasonal sensitivity (Ashman et al. 1999). This multi‐domain approach reflects the interconnected nature of sleep, mood, and circadian systems (Yuh et al. 2026) and aligns with the comprehensive phenotyping framework of the SOMDAY study (Lee et al. 2025).

We investigated this question using data from the SOMDAY deep phenotyping study (Lee et al. 2025). We employed a 2 × 2 factorial design crossing sleep status (insomnia vs. control) with bipolarity (K‐MDQ ≥ 7 vs. < 7) to examine the independent and interactive effects across 14 validated clinical scales spanning sleep, mood, chronotype, resilience, fatigue, substance use, and seasonality domains.

We tested three hypotheses: (H1) sleep Status would show significant main effects across multiple clinical domains; (H2) Bipolarity would function as an independent correlate of elevated clinical severity across both sleep and non‐sleep domains—this represents the core contribution of the study, and (H3) exploratory interaction effects might reveal domain‐specific patterns where insomnia and high bipolarity produce disproportionately elevated symptom profiles.

## 2 Methods

### 2.1 Study Design and Participants

This study used data from the SOMDAY deep phenotyping study, a prospective observational study designed to comprehensively characterize insomnia through multimodal assessment (Lee et al. 2025). The study employed a parallel‐cohort design with two groups: an insomnia group and a normal sleeper control group. Participants aged 19–70 years were recruited from the local community through online platforms and campus bulletin boards between March 2023 and October 2024.

The insomnia group included individuals reporting subjective insomnia symptoms at least three times per week over the preceding three months, with an Insomnia Severity Index (ISI) score of ≥ 15. The normal sleeper control group included individuals with insomnia symptoms fewer than three times per week, ISI < 8, and an average sleep duration of ≥ 6 h per night. Exclusion criteria included other primary sleep disorders, current major psychiatric diagnoses (major depression, bipolar disorder, schizophrenia, or severe substance use disorder), cognitive impairment, neurological disorders, shift work, and current use of hypnotic medications (Lee et al. 2025).

A total of 338 participants were enrolled (252 insomnia, 86 controls). All participants provided written informed consent. The study was approved by the Institutional Review Board of Korea University Anam Hospital (IRB No. 2022AN0587) and conducted in accordance with the Declaration of Helsinki. Missing data rates on the 14 primary clinical scales were minimal (< 2% per scale); cases with missing values were excluded on a per‐analysis basis.

### 2.2 Measures

#### 2.2.1 Sleep‐Related Assessments

Sleep quality was assessed using the Pittsburgh Sleep Quality Index (PSQI; global score 0–21, >5 indicating poor sleep) (Buysse et al. 1989). Daytime sleepiness was measured by the Epworth Sleepiness Scale (ESS; 0–24, >10 indicating excessive sleepiness) (Johns 1991). Maladaptive sleep cognitions were assessed with the Dysfunctional Beliefs and Attitudes about Sleep‐16 (DBAS‐16; mean score 0–10) (Morin et al. 2007). Bedtime procrastination was measured by the Bedtime Procrastination Scale (BPS; 9–45) (Kroese et al. 2014). Vulnerability to stress‐reactive insomnia was assessed using the Ford Insomnia Response to Stress Test (FIRST; 9–36) (Drake et al. 2004).

#### 2.2.2 Psychological and Mental Health Assessments

Depression was measured by the Patient Health Questionnaire‐9 (PHQ‐9; 0–27) (Kroenke et al. 2001). Anxiety was assessed using the Generalized Anxiety Disorder‐7 (GAD‐7; 0–21) (Spitzer et al. 2006). Anxiety sensitivity was evaluated with the Body Sensations Questionnaire (BSQ) (Chambless et al. 1984). Psychological resilience was measured by the Korean Resilience Quotient‐53 (KRQ‐53; 53–265) (Kim 2011). Bipolar spectrum screening was conducted using the K‐MDQ (Jon et al. 2009).

#### 2.2.3 General Health and Lifestyle Assessments

Chronotype was determined using the Composite Scale of Morningness (CSM; 13–55) (Smith et al. 1989). Circadian rhythm disturbance was quantified by the Korean version of the Biological Rhythms Interview of Assessment in Neuropsychiatry (K‐BRIAN; 18–72) (C. H. Cho et al. 2018). Fatigue was assessed using the Multidimensional Fatigue Scale (MFS; 19–133) (Schwartz et al. 1993). Alcohol use was screened with the Korean version of the Alcohol Use Disorders Identification Test (AUDIT‐K; 0–40) (Chang et al. 2016). Seasonal mood variation was assessed using the Seasonal Pattern Assessment Questionnaire Global Seasonality Score (SPAQ GSS; 0–24) (Rosenthal 1984).

### 2.3 Sleep Status Classification

Participants were classified into insomnia and normal sleeper control groups based on predefined criteria described in the SOMDAY study protocol (Lee et al. 2025). The insomnia group required: (a) subjective insomnia symptoms at least three times per week over the preceding three months, and (b) an Insomnia Severity Index (ISI) score of ≥ 15. The normal sleeper control group required (a) insomnia symptoms fewer than three times per week, (b) ISI < 8, and (c) an average sleep duration of ≥ 6 h per night. This binary classification served as the Sleep Status factor in the 2 × 2 factorial design.

### 2.4 Bipolarity Classification

Bipolarity was operationalized using the K‐MDQ Section 1 symptom count. The K‐MDQ is a 3‐section screening instrument: Section 1 assesses 13 lifetime manic/hypomanic symptoms (yes/no), Section 2 asks whether endorsed symptoms co‐occurred, and Section 3 evaluates functional impairment (Jon et al. 2009). In the present study, we used only the Section 1 symptom count rather than the full three‐section algorithm, consistent with dimensional approaches that treat bipolarity as a continuous trait rather than a categorical diagnosis (Angst et al. 2012). This approach has been used in prior research examining bipolar spectrum features in non‐clinical and sleep‐disordered populations, where functional impairment criteria may underestimate subthreshold bipolarity (Cho et al. 2016). Participants endorsing ≥ 7 of 13 lifetime manic/hypomanic symptoms were classified as high bipolarity (K‐MDQ High), and those endorsing < 7 as low bipolarity (K‐MDQ Low). This cutoff is consistent with the Section 1 threshold established in the original K‐MDQ validation (Jon et al. 2009) and yielded a high bipolarity prevalence of 38.2% (*n* = 129). Sensitivity analyses using alternative cutoffs (≥ 8 and ≥ 9) were conducted to assess the robustness of findings.

### 2.5 Statistical Analysis

#### 2.5.1 Primary Factorial Analysis (H1, H2)

The primary analysis employed a 2 × 2 between‐subjects factorial design. Two‐way analyses of covariance (ANCOVA) were conducted for each of the 14 clinical scales using Type II sums of squares, with age and sex as covariates. Age and sex were selected as covariates because they showed significant between‐group differences (Table 1) and are established confounders in mood and sleep research. Education level and body mass index (BMI) were considered as additional covariates; supplementary analyses including these variables confirmed that nearly all primary results remained unchanged, with only FIRST and MFS bipolarity effects becoming marginal (pFDR = 0.056 and 0.080, respectively) (Table S1).

**TABLE 1:** Demographic and clinical characteristics of participants.

Variable	Control/Low (*n* = 62)	Control/High (*n* = 24)	Insomnia/Low (*n* = 147)	Insomnia/High (*n* = 105)
Age (years)	24.4 ± 3.9	24.8 ± 3.9	33.3 ± 11.0	29.2 ± 8.7
Female, n (%)	43 (69.4)	10 (41.7)	102 (69.4)	64 (61.0)
BMI (kg/m^2^)	21.6 ± 3.3	22.1 ± 2.2	22.6 ± 3.1	23.0 ± 4.0

*Note*: Values are presented as mean ± SD or n (%). K‐MDQ, Korean version of the Mood Disorder Questionnaire. High bipolarity = K‐MDQ ≥ 7; Low bipolarity = K‐MDQ < 7.

#### 2.5.2 Multiple Comparison Correction and Effect Sizes

The Benjamini–Hochberg false discovery rate (FDR) procedure was applied separately for each effect type (sleep status, bipolarity, interaction) across the 14 primary scales. This approach was chosen because each effect type tests a distinct hypothesis (H1–H3), and applying FDR within each family preserves the interpretability of effect‐specific conclusions while controlling false discoveries within each research question. FDR‐corrected *p* < 0.05 was considered significant. Effect sizes were reported as partial eta‐squared (η^2^p), with 0.01, 0.06, and 0.14 as small, medium, and large benchmarks (Cohen 1988).

#### 2.5.3 Exploratory and Sensitivity Analyses (H3)

Exploratory subdomain analyses were conducted for multi‐component scales (PSQI C1–C7, KRQ‐53, K‐BRIAN, and MFS subdomains) and reported with uncorrected *p*‐values. Homogeneity of regression slopes was assessed for each scale and found to be satisfactory. Levene's test for homogeneity of variance was conducted for each scale; results indicated acceptable homogeneity (all *p* > 0.01, given unequal cell sizes). Sensitivity analyses used rank‐transformed ANCOVA (Conover and Iman 1982) to assess robustness to normality violations. For interaction effects with uncorrected *p* < 0.10, simple effects analyses were performed. Post‐hoc power analysis indicated that with *N* = 338 and the observed cell sizes, the study had approximately 80% power to detect interaction effects of η^2^p ≥ 0.023 (small‐to‐medium) at α = 0.05. The smallest cell (Control/High: *n* = 24) may have limited power for detecting smaller interaction effects; accordingly, the null interaction results should be interpreted with caution. Analyses were conducted using Python (scipy, statsmodels).

## 3 Results

### 3.1 Participant Characteristics

Table 1 presents demographic characteristics. Of the 338 participants, 209 (61.8%) were classified as K‐MDQ Low and 129 (38.2%) as K‐MDQ High. The insomnia group was significantly older than controls (*F* = 18.00, *p* < 0.001), and the sex distribution differed across groups (χ^2^ = 8.05, *p* = 0.045). Age and sex were included as covariates in all subsequent analyses.

### 3.2 Main Effects of Sleep Status

**FIGURE 1:**
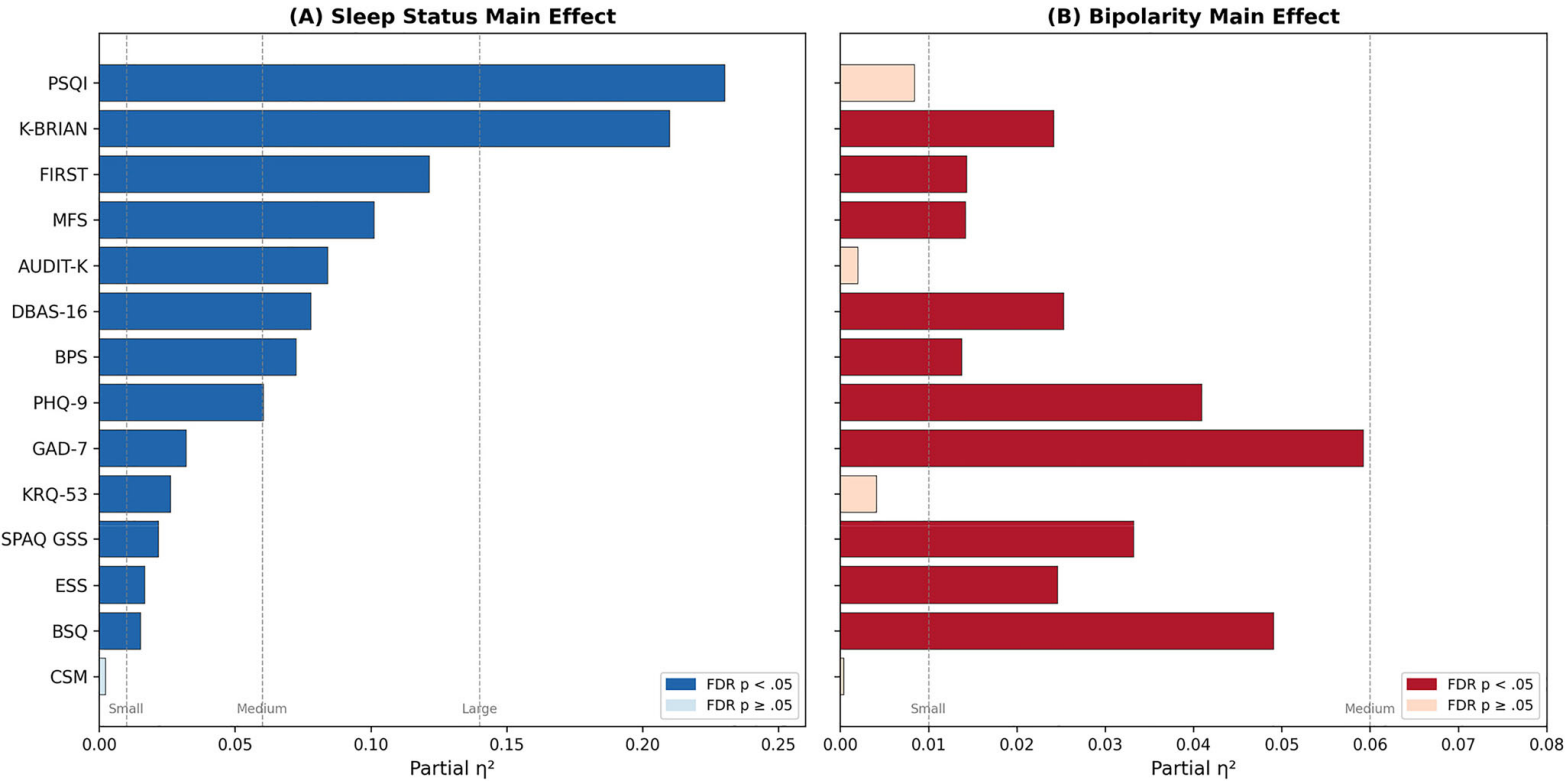
Comparison of effect sizes (partial η^2^) for sleep status (A) and bipolarity (B) main effects across 14 clinical scales. Dark blue/red bars indicate FDR‐corrected *p* < 0.05; light bars indicate non‐significant effects. Dashed lines represent Cohen's (1988) conventional benchmarks (small = 0.01, medium = 0.06, large = 0.14). PSQI, Pittsburgh Sleep Quality Index; ESS, Epworth Sleepiness Scale; DBAS‐16, Dysfunctional Beliefs and Attitudes about Sleep‐16; BPS, Bedtime Procrastination Scale; FIRST, Ford Insomnia Response to Stress Test; PHQ‐9, Patient Health Questionnaire‐9; GAD‐7, Generalized Anxiety Disorder‐7; BSQ, Body Sensations Questionnaire; KRQ‐53, Korean Resilience Quotient‐53; CSM, Composite Scale of Morningness; K‐BRIAN, Korean Biological Rhythms Interview of Assessment in Neuropsychiatry; MFS, Multidimensional Fatigue Scale; AUDIT‐K, Korean Alcohol Use Disorders Identification Test; SPAQ GSS, Seasonal Pattern Assessment Questionnaire Global Seasonality Score.

Sleep status exerted significant main effects on 13 of 14 clinical scales after FDR correction (Table 2, Figure 1). The only non‐significant scale was chronotype (CSM, pFDR = 0.374). The largest effects were observed for PSQI (η2p = 0.230, pFDR < 0.001), K‐BRIAN (η2p = 0.210, pFDR < 0.001), FIRST (η2p = 0.121, pFDR < 0.001), and MFS (η2p = 0.101, pFDRSleep status exerted significant main effects on 13 of 14 clinical scales after FDR correction (Table 2, Figure 1). The only non‐significant scale was chronotype (CSM, pFDR = 0.374). The largest effects were observed for PSQI (η2p = 0.230, pFDR < 0.001), K‐BRIAN (η2p = 0.210, pFDR < 0.001), FIRST (η2p = 0.121, pFDR < 0.001), and MFS (η2p = 0.101, pFDR < 0.001). Moderate effects were found for AUDIT‐K (η2p = 0.084), DBAS‐16 (η2p = 0.078), BPS (η2p = 0.073), and PHQ‐9 (η2p = 0.060). The insomnia group scored consistently higher (more severe) on all sleep, mood, and fatigue measures and lower on AUDIT‐K compared to controls (Table 3).

**TABLE 2:** Two‐way ANCOVA results for the 14 primary clinical scales.

Scale	N	F (Sleep)	*p*_FDR_	η^2^_p_	F (Bipol)	*p*_FDR_	η^2^_p_	F (Int)	*p*_FDR_
PSQI	337	99.01	**<0.001**	0.230	2.81	0.120	0.008	1.83	0.850
ESS	337	5.64	**0.021**	0.017	8.35	**0.009**	0.025	0.47	0.850
DBAS‐16	337	28.01	**<0.001**	0.078	8.60	**0.009**	0.025	0.52	0.850
BPS	336	25.90	**<0.001**	0.073	5.53	**0.034**	0.016	0.05	0.911
FIRST	337	45.76	**<0.001**	.121	4.80	**0.042**	0.014	0.01	0.911
PHQ‐9	337	21.28	**<0.001**	0.060	14.13	**<0.001**	0.041	1.08	0.850
GAD‐7	337	10.95	**0.002**	0.032	20.85	**<0.001**	0.059	0.40	0.850
BSQ	337	5.10	**0.026**	0.015	17.09	**<0.001**	0.049	0.73	0.850
KRQ‐53	337	8.94	**0.004**	0.026	1.37	0.284	0.004	0.05	0.911
CSM	337	0.79	0.374	0.002	0.13	0.714	0.000	0.02	0.911
K‐BRIAN	337	87.98	**<0.001**	0.210	8.20	**0.009**	0.024	0.36	0.850
MFS	337	37.27	**<0.001**	0.101	4.77	**0.042**	0.014	4.42	0.508
AUDIT‐K	337	30.38	**<0.001**	0.084	0.66	0.449	0.002	0.08	0.911
SPAQ GSS	337	7.37	**0.009**	0.022	11.39	**0.003**	0.033	1.36	0.850

*Notes*: Covariates: age, sex.

Bold p_FDR_ values indicate FDR‐corrected p < 0.05.

η^2^p = partial eta‐squared. Int = Sleep Status × Bipolarity interaction.

Abbreviations: AUDIT‐K = Korean Alcohol Use Disorders Identification Test; BPS = Bedtime Procrastination Scale; BSQ = Body Sensations Questionnaire; CSM = Composite Scale of Morningness; DBAS‐16 = Dysfunctional Beliefs and Attitudes about Sleep‐16; ESS = Epworth Sleepiness Scale; FIRST = Ford Insomnia Response to Stress Test; GAD‐7 = Generalized Anxiety Disorder‐7; K‐BRIAN = Korean Biological Rhythms Interview of Assessment in Neuropsychiatry; KRQ‐53 = Korean Resilience Quotient‐53; MFS = Multidimensional Fatigue Scale; PHQ‐9 = Patient Health Questionnaire‐9; PSQI = Pittsburgh Sleep Quality Index; SPAQ GSS = Seasonal Pattern Assessment Questionnaire Global Seasonality Score.

**TABLE 3:** Descriptive statistics for the 14 clinical scales by group (mean ± SD).

Scale	Control/Low (*n =* 62)	Control/High (*n =* 24)	Insomnia/Low (*n =* 147)	Insomnia/High (*n =* 105)
PSQI	5.2 ± 1.7	6.3 ± 2.1	8.8 ± 2.7	9.1 ± 2.7
ESS	6.5 ± 3.3	7.2 ± 3.7	7.3 ± 3.9	8.8 ± 3.9
DBAS‐16	4.4 ± 1.3	4.6 ± 1.5	5.2 ± 1.5	5.8 ± 1.5
BPS	24.9 ± 6.3	26.3 ± 3.9	27.3 ± 5.5	29.2 ± 5.4
FIRST	17.7 ± 5.0	18.5 ± 5.1	23.4 ± 6.4	24.4 ± 5.7
PHQ‐9	3.2 ± 3.3	5.8 ± 5.5	5.9 ± 4.2	7.6 ± 5.0
GAD‐7	1.8 ± 2.6	2.9 ± 3.5	3.1 ± 3.7	5.2 ± 4.7
BSQ	32.3 ± 13.3	34.5 ± 11.0	35.0 ± 12.0	41.6 ± 14.0
KRQ‐53	178.5 ± 28.8	175.3 ± 17.5	187.0 ± 25.7	184.9 ± 22.8
CSM	20.5 ± 5.2	20.5 ± 4.7	19.9 ± 4.5	20.6 ± 4.9
K‐BRIAN	24.9 ± 12.4	26.2 ± 13.6	36.1 ± 9.3	40.2 ± 9.8
MFS	77.0 ± 17.8	72.4 ± 17.8	85.8 ± 18.8	93.8 ± 18.1
AUDIT‐K	15.9 ± 13.1	16.0 ± 13.3	7.6 ± 8.3	7.5 ± 7.5
SPAQ GSS	5.2 ± 4.3	8.0 ± 6.4	3.9 ± 4.2	5.4 ± 5.4

Abbreviations: K‐MDQ High = K‐MDQ ≥ 7; Low = K‐MDQ < 7. For abbreviations, see Table 2 footnote.

### 3.3 Main Effects of Bipolarity

**FIGURE 2:**
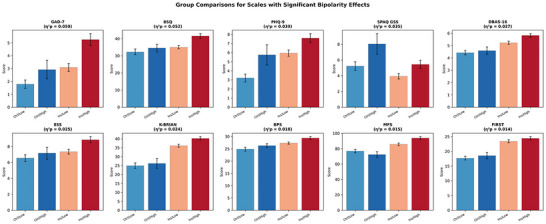
Group comparisons for the 10 clinical scales with significant bipolarity main effects (FDR‐corrected *p* < 0.05). Bars represent group means; error bars represent ± 1 standard error. Ctrl, Control; Ins, Insomnia; Low/High, K‐MDQ bipolarity classification (K‐MDQ < 7 vs. ≥ 7). η^2^p, partial eta‐squared from the bipolarity main effect in two‐way ANCOVA (covariates: age, sex). K‐MDQ, Korean version of the Mood Disorder Questionnaire. For scale abbreviations, see Figure 1 legend.

Bipolarity exerted significant independent main effects on 10 of 14 clinical scales after FDR correction (Table 2, Figure 1). The strongest bipolarity effects were observed for GAD‐7 (η2p = 0.059, pFDR < 0.001), BSQ (η2p = 0.049, pFDR < 0.001), PHQ‐9 (η2p = 0.041, pFDR < 0.001), and SPAQ GSS (η2p = 0.033, pFDR = 0.003). Additional significant effects included DBAS‐16 (η2p = 0.025, pFDR = 0.009), ESS (η2p = 0.025, pFDR = 0.009), K‐BRIAN (η2p = 0.024, pFDRBipolarity exerted significant independent main effects on 10 of 14 clinical scales after FDR correction (Table 2, Figure 1). The strongest bipolarity effects were observed for GAD‐7 (η2p = 0.059, pFDR < 0.001), BSQ (η2p = 0.049, pFDR < 0.001), PHQ‐9 (η2p = 0.041, pFDR < 0.001), and SPAQ GSS (η2p = 0.033, pFDR = 0.003). Additional significant effects included DBAS‐16 (η2p = 0.025, pFDR = 0.009), ESS (η2p = 0.025, pFDR = 0.009), K‐BRIAN (η2p = 0.024, pFDR = 0.009), MFS (η2p = 0.014, pFDR = 0.042), FIRST (η2p = 0.014, pFDR = 0.042), and BPS (η2p = 0.016, pFDR = 0.034). In all cases, the K‐MDQ High group exhibited more severe profiles (Table 3, Figure 2).

Four scales did not show significant bipolarity effects: PSQI (*p* = 0.095, pFDR = 0.120), KRQ‐53 (*p* = 0.243, pFDR = 0.284), CSM (*p* = 0.714, pFDR = 0.714), and AUDIT‐K (*p* = 0.417, pFDR = 0.449).

### 3.4 Interaction Effects

**FIGURE 3:**
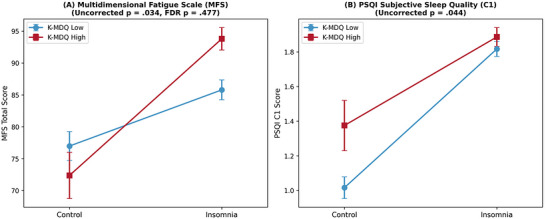
Interaction plots for scales showing exploratory interaction trends. (A) Multidimensional Fatigue Scale (MFS): the crossing pattern suggests a possible synergistic effect in the insomnia group, where high bipolarity (K‐MDQ ≥ 7) was associated with greater fatigue in patients but paradoxically lower fatigue in controls (uncorrected *p* = 0.036; FDR‐corrected *p* = 0.508). (B) Pittsburgh Sleep Quality Index Component 1 (PSQI C1; Subjective Sleep Quality): a similar interaction pattern was observed (uncorrected *p* = 0.044). Error bars represent ± 1 standard error. K‐MDQ, Korean version of the Mood Disorder Questionnaire; PSQI, Pittsburgh Sleep Quality Index; MFS, Multidimensional Fatigue Scale.

No interaction effects survived FDR correction (Table 2). Uncorrected interaction p‐values ranged from 0.036 (MFS) to 0.911 (FIRST), with only the MFS reaching nominal significance; all other interaction p‐values exceeded 0.10. The absence of significant interactions indicates that the effects of bipolarity are predominantly additive rather than synergistic with insomnia status, though the limited power for interaction detection (see 2. Methods) warrants cautious interpretation. One exploratory finding warrants mention: the MFS showed an uncorrected interaction trend (*F =* 4.42, *p* = 0.036, η^2^p = 0.013), though this did not survive correction (pFDR = 0.508). Simple effects analysis suggested that the bipolarity effect on fatigue was more pronounced in the insomnia group (Insomnia/Low: 85.8 ± 18.8 vs. Insomnia/High: 93.8 ± 18.1) compared to controls (Control/Low: 77.0 ± 17.8 vs. Control/High: 72.4 ± 17.8), where high bipolarity was paradoxically associated with lower fatigue (Figure 3A). A similar interaction pattern was observed for PSQI C1 (Subjective Sleep Quality; uncorrected p = 0.044), where high bipolarity was associated with relatively poorer subjective quality in the insomnia group but not in controls (Figure 3B).

### 3.5 Exploratory Subdomain Analyses

Among PSQI components, C7 (Daytime Dysfunction) showed both a sleep status effect (*p* = 0.001) and a bipolarity effect (*p* < 0.001), while C1 (Subjective Quality) showed a nominal, uncorrected interaction (*p* = 0.044). Among K‐BRIAN subdomains, the social rhythm domain showed the strongest bipolarity effect (*p* = 0.004), followed by the activity domain (*p* = 0.019). For KRQ‐53, the Self‐Regulation subdomain showed a selective bipolarity effect (*p* = 0.027) not apparent in the total score. Among MFS subdomains, mental fatigue showed both a bipolarity effect (*p* = 0.023) and an interaction trend (*p* = 0.048).

### 3.6 Sensitivity Analyses

Rank‐transformed ANCOVA confirmed the robustness of all parametric findings. All scales reaching FDR significance in the primary analysis maintained significance in the rank‐transformed analysis, and no new effects emerged. This concordance supports the validity of findings across scales with varying distributional properties.

## 4 Discussion

This study examined the independent and interactive contributions of insomnia status and bipolarity to clinical phenotypes across 14 validated clinical scales. Using clinical questionnaire data from the SOMDAY deep phenotyping study (Lee et al. 2025), we found that bipolarity, as indexed by the K‐MDQ, exerts statistically independent main effects on multiple clinical dimensions—reflecting greater psychological distress, anxiety sensitivity, and circadian vulnerability—in a pattern that is additive to, rather than contingent upon, insomnia status.

Bipolarity showed significant main effects across 10 of 14 scales after FDR correction, even after controlling for age, sex, and insomnia status in the factorial ANCOVA framework. Critically, the term ‘independent’ as used throughout this paper refers to statistical independence within the factorial design: bipolarity main effects are estimated after partialing out the variance attributable to sleep status, meaning that the bipolarity‐related elevations in clinical severity exist over and above any effects of insomnia. The consistency of these effects across both insomnia and control groups further supports the conceptualization of bipolarity as a transdiagnostic dimension that transcends the insomnia–normal sleep boundary.

The strongest bipolarity effects were in the mood and anxiety domain. GAD‐7 (η^2^p = 0.059), BSQ (η^2^p = 0.049), and PHQ‐9 (η^2^p = 0.041) showed the largest bipolarity effects. Although small‐to‐moderate by conventional benchmarks, these effects were consistent across multiple domains and survived FDR correction (Cohen 1988). These findings align with known associations between bipolar spectrum features and emotional dysregulation (Angst et al. 2012; Harvey 2008). The BSQ finding is particularly notable, as anxiety sensitivity—the fear of autonomic sensations—has been identified as a vulnerability factor for both anxiety disorders and sleep disturbance (Chambless et al. 1984; Khalsa et al. 2018). Elevated anxiety sensitivity in high‐bipolarity individuals may link bipolarity to sleep difficulties through heightened interoceptive awareness.

The significant bipolarity effect on SPAQ GSS (η2p = 0.033) aligns with the established connection between bipolar spectrum conditions and seasonal mood sensitivity (Ashman et al. 1999). Combined with bipolarity effects on K‐BRIAN (η2p = 0.024) and DBAS‐16 (η2p = 0.025), this pattern points to a broad circadian‐cognitive‐affective cluster associated with bipolarity extending beyond primary sleep disturbance.

The pattern of non‐significant bipolarity effects is informative. PSQI, the primary sleep quality measure and the scale most strongly associated with insomnia status (η^2^p = 0.230), was not independently associated with bipolarity (*p* = 0.095). It should be noted that the large sleep status main effect on PSQI is partly expected given that ISI (the grouping variable) and PSQI are highly correlated (typically *r* > 0.70), introducing a degree of criterion contamination. Nevertheless, the dissociation between the strong sleep status effect and the null bipolarity effect on PSQI suggests that bipolarity is more closely linked to the surrounding psychological and circadian landscape than to the core sleep quality disturbance.

KRQ‐53 (resilience) was similarly unaffected by bipolarity (*p* = 0.243), suggesting that resilience may be more closely associated with the chronic burden of insomnia than with bipolar spectrum features. CSM (chronotype) was unaffected by either factor, indicating stable morningness‐eveningness preferences. AUDIT‐K showed only a sleep status effect—lower consumption in the insomnia group, likely reflecting exclusion criteria or behavioral self‐regulation around sleep—with no bipolarity modulation.

The absence of significant interaction effects after FDR correction is consistent with an additive model in which bipolarity and insomnia contribute independently to clinical severity. In a factorial ANCOVA framework, the lack of interaction indicates that the bipolarity‐related increment in symptom severity is consistent across both insomnia and control groups, rather than being contingent on sleep status. Notably, the observation that many of the same scales show significant effects of both sleep status and bipolarity does not indicate redundancy; rather, it reflects additive contributions—bipolarity adds a consistent layer of clinical burden on top of insomnia‐related effects. This additive pattern is precisely what supports the clinical utility of bipolar spectrum screening in insomnia populations: bipolarity identifies an additional, non‐overlapping source of symptom severity. However, given the limited statistical power for interaction effects in the present sample, particularly with the smallest cell at *n =* 24, the possibility of true but undetected interactions cannot be excluded. Larger studies with balanced cell sizes are needed to definitively evaluate synergistic effects.

The exploratory MFS interaction (*p* = 0.036 uncorrected, η2p = 0.013) is biologically plausible: chronic sleep disruption combined with mood instability may produce synergistic fatigue exceeding the sum of individual effects. However, this did not survive FDR correction and should be treated as hypothesis‐generating.

These findings complement previous SOMDAY reports. Matsushita et al. (2026) demonstrated that smartphone overuse among insomnia participants was independently associated with greater circadian rhythm disruption (K‐BRIAN), more severe insomnia, and elevated depression and anxiety after covariate adjustment. Jeong et al. (2025) showed that machine learning models using digital phenotypes could predict restless legs syndrome within this cohort. Kim et al. (2026) identified heart rate circadian phase shifts and hyperarousal signatures as interpretable wearable‐based digital phenotypes of insomnia, further demonstrating the utility of multimodal assessment in this population. The present findings suggest that bipolarity constitutes an additional dimension of clinical heterogeneity that crosscuts the insomnia–control boundary and influences phenotypes through a distinct mood vulnerability pathway.

These findings have several clinical implications that warrant further investigation. First, the independent association of bipolarity with elevated anxiety, depression, anxiety sensitivity, and circadian disruption supports the potential utility of routine bipolar spectrum screening (e.g., K‐MDQ) in sleep medicine settings. Identifying high‐bipolarity individuals may inform treatment planning; for example, future interventional research could examine whether augmenting standard cognitive‐behavioral therapy for insomnia (CBT‐I) with mood‐focused components such as behavioral activation, emotion regulation strategies, and circadian stabilization protocols improves outcomes in this subgroup (Ashman et al. 1999; Harvey 2008). The elevated anxiety sensitivity observed in high‐bipolarity individuals (BSQ η^2^p = 0.049) also warrants attention, as anxiety sensitivity can maintain insomnia through hyperarousal and interoceptive hypervigilance (Khalsa et al. 2018); whether targeted interoceptive exposure techniques benefit this subgroup remains to be tested.

Second, bipolarity was associated with greater clinical burden even among normal sleepers, suggesting that individuals presenting with subclinical sleep complaints alongside elevated mood symptoms may benefit from systematic evaluation for bipolar spectrum features. This is particularly relevant for primary care and community screening, where sleep complaints are common but bipolarity is rarely assessed. Third, the additive effect pattern—where bipolarity and insomnia each contribute independently to clinical severity—provides assessment guidance: these two dimensions should be evaluated separately, as their clinical contributions are largely non‐overlapping. This is consistent with a dimensional approach to assessment, aligning with emerging frameworks in precision psychiatry that emphasize individual risk profiling (Angst et al. 2012). Fourth, the selective non‐significance of bipolarity on PSQI suggests that overall sleep quality measures alone may not capture the added clinical complexity associated with high bipolarity; multidimensional assessment batteries that include mood, anxiety, and circadian rhythm measures are needed for comprehensive evaluation.

### 4.1 Limitations

Several limitations should be noted. First, the cross‐sectional design precludes causal inference; whether bipolarity drives clinical severity or shared vulnerabilities produce both elevated K‐MDQ scores and symptoms remains unclear. Longitudinal studies are needed. Second, the K‐MDQ is a screening instrument, not a diagnostic tool; the high bipolarity group likely includes individuals across a spectrum from normal mood variability to undiagnosed bipolar spectrum conditions. Moreover, we used only the Section 1 symptom count (≥ 7 of 13 items) rather than the full three‐section K‐MDQ algorithm (which also requires symptom co‐occurrence and functional impairment), which may have overestimated bipolarity prevalence. Sensitivity analyses using stricter cutoffs (≥ 8 and ≥ 9) confirmed that the core findings remained robust (Table S2). Third, the sample was recruited from a Korean community, potentially limiting generalizability to other populations. Fourth, ISI was used as a grouping criterion and was therefore excluded from outcome variables; however, the correlation between ISI and PSQI (a primary outcome) introduces a degree of criterion contamination that inflates the sleep status main effect on PSQI. Fifth, some cell sizes were modest (Control/High: *n =* 24), which limited power for detecting interaction effects (η^2^p < 0.023); the null interaction results should therefore be treated as inconclusive rather than definitive. Finally, although the SOMDAY study collected digital phenotyping and neuroimaging data, the present analysis was limited to clinical scale data; future studies should integrate these additional modalities to provide a more comprehensive characterization.

## 5 Conclusion

Bipolarity, as indexed by the K‐MDQ, exerts statistically independent main effects associated with greater psychological distress, anxiety sensitivity, circadian vulnerability, and fatigue, operating additively with insomnia status across both insomnia patients and normal sleepers. The additive effect pattern indicates that bipolarity and insomnia represent distinct but complementary dimensions of clinical risk, each contributing independently to symptom severity. These findings support integrating bipolar spectrum screening into sleep medicine practice and highlight the potential for bipolarity‐informed treatment stratification.

## Author Contributions

**Sujin Kim**: writing – original draft. **Seung Pil Pack**: data curation. **Heon‐Jeong Lee**: supervision. **Chul‐Hyun Cho**: conceptualization, formal analysis, funding acquisition, investigation, writing – original draft, writing – review and editing.

## Funding

This study was supported by the National Research Foundation (NRF) of Korea (grant number: NRF‐2021R1A5A8032895, NRF‐2022M3C1B6080866, RS‐2026‐25523612, and RS‐2026‐25471696). The funders had no role in study design, data collection, analysis, interpretation, manuscript preparation, or the decision to submit the article for publication.

## Ethics Statement

This study was approved by the Institutional Review Board of Korea University Anam Hospital (IRB No. 2022AN0587). All procedures were conducted in accordance with the Declaration of Helsinki. Written informed consent was obtained from all participants prior to enrollment.

## Conflicts of Interest

The authors declare no conflicts of interest.

## Supporting information




**Supplementary Tables**: brb371642‐sup‐0001‐TableS1‐S2.docx

## Data Availability

Data supporting the findings of this study are available upon request from the corresponding authors. The data are not publicly available due to privacy concerns regarding participants’ professional or privacy‐sensitive information and institutional review board requirements for confidentiality. Researchers interested in accessing de‐identified data for verification or secondary analysis may contact the corresponding author with a detailed research proposal. Data sharing, if approved, will be subject to appropriate institutional review and data use agreements.

## References

[brb371642-bib-0001] Angst, J. , A. Gamma , C. L. Bowden , et al. 2012. “Diagnostic Criteria for Bipolarity Based on an International Sample of 5,635 Patients With DSM‐IV Major Depressive Episodes.” European Archives of Psychiatry and Clinical Neuroscience 262, no. 1: 3–11. 10.1007/s00406-011-0228-0. 21818629

[brb371642-bib-0002] Ashman, S. B. , T. H. Monk , D. J. Kupfer , et al. 1999. “Relationship Between Social Rhythms and Mood in Patients With Rapid Cycling Bipolar Disorder.” Psychiatry Research 86, no. 1: 1–8. 10.1016/s0165-1781(99)00019-0. 10359478

[brb371642-bib-0003] Buysse, D. J. , C. F. Reynolds 3rd , T. H. Monk , S. R. Berman , and D. J. Kupfer . 1989. “The Pittsburgh Sleep Quality Index: A New Instrument for Psychiatric Practice and Research.” Psychiatry Research 28, no. 2: 193–213. 10.1016/0165-1781(89)90047-4. 2748771

[brb371642-bib-0004] Chambless, D. L. , G. C. Caputo , P. Bright , and R. Gallagher . 1984. “Assessment of Fear of Fear in Agoraphobics: The Body Sensations Questionnaire and the Agoraphobic Cognitions Questionnaire.” Journal of Consulting and Clinical Psychology 52, no. 6: 1090–1097. 10.1037//0022-006x.52.6.1090. 6520279

[brb371642-bib-0005] Chang, J. W. , J. S. Kim , J. G. Jung , S. S. Kim , S. J. Yoon , and H. S. Jang . 2016. “Validity of Alcohol Use Disorder Identification Test‐Korean Revised Version for Screening Alcohol Use Disorder According to Diagnostic and Statistical Manual of Mental Disorders, Fifth Edition Criteria.” Korean Journal of Family Medicine 37, no. 6: 323. 10.4082/kjfm.2016.37.6.323. 27900069 PMC5122663

[brb371642-bib-0006] Cho, C. H. , S. Y. Jung , F. Kapczinski , A. R. Rosa , and H. J. Lee . 2018. “Validation of the Korean Version of the Biological Rhythms Interview of Assessment in Neuropsychiatry.” Psychiatry Investigation 15, no. 12: 1115–1120. 10.30773/pi.2018.10.21.1. 30602104 PMC6318494

[brb371642-bib-0007] Cho, C.‐H. , J.‐H.o Moon , H.o‐K. Yoon , et al. 2016. “Molecular Circadian Rhythm Shift due to Bright Light Exposure Before Bedtime Is Related to Subthreshold Bipolarity.” Scientific Reports 6, no. 1: 31846. 10.1038/srep31846. 27545669 PMC4992827

[brb371642-bib-0008] Cohen, J. 1988. Statistical Power Analysis for the Behavioral Sciences, 2nd ed. Lawrence Erlbaum Associates.

[brb371642-bib-0009] Conover, W. J. , and R. L. Iman . 1982. “Analysis of Covariance Using the Rank Transformation.” Biometrics 38, no. 3: 715. 10.2307/2530051. 7171697

[brb371642-bib-0010] Drake, C. , G. Richardson , T. Roehrs , H. Scofield , and T. Roth . 2004. “Vulnerability to Stress‐Related Sleep Disturbance and Hyperarousal.” Sleep 27, no. 2: 285–291. 10.1093/sleep/27.2.285. 15124724

[brb371642-bib-0011] Giglio, L. M. , P. V. S. Magalhães , N. S. Kapczinski , J. C. Walz , and F. Kapczinski . 2010. “Functional Impact of Biological Rhythm Disturbance in Bipolar Disorder.” Journal of Psychiatric Research 44, no. 4: 220–223. 10.1016/j.jpsychires.2009.08.003. 19758600

[brb371642-bib-0012] Harvey, A. G. 2008. “Sleep and Circadian Rhythms in Bipolar Disorder: Seeking Synchrony, Harmony, and Regulation.” American Journal of Psychiatry 165, no. 7: 820–829. 10.1176/appi.ajp.2008.08010098. 18519522

[brb371642-bib-0013] Harvey, A. G. 2011. “Sleep and Circadian Functioning: Critical Mechanisms in the Mood Disorders?” Annual Review of Clinical Psychology 7: 297–319. 10.1146/annurev-clinpsy-032210-104550. 21166537

[brb371642-bib-0014] Jeong, J. , Y. Jeon , H. Kim , et al. 2025. “Machine Learning‐Based Prediction of Restless Legs Syndrome Using Digital Phenotypes From Wearables and Smartphone Data.” Scientific Reports 15, no. 1: 16349. 10.1038/s41598-025-01215-8. 40348809 PMC12065804

[brb371642-bib-0015] Johns, M. W. 1991. “A New Method for Measuring Daytime Sleepiness: the Epworth Sleepiness Scale.” Sleep 14, no. 6: 540–545. 10.1093/sleep/14.6.540. 1798888

[brb371642-bib-0016] Jon, D. I. , N. Hong , B. H. Yoon , et al. 2009. “Validity and Reliability of the Korean Version of the Mood Disorder Questionnaire.” Comprehensive Psychiatry 50, no. 3: 286–291. 10.1016/j.comppsych.2008.07.008. 19374975

[brb371642-bib-0017] Khalsa, S. S. , R. Adolphs , O. G. Cameron , et al. 2018. “Interoception and Mental Health: A Roadmap.” Biol Psychiatry Cogn Neurosci Neuroimaging 3, no. 6: 501–513. 10.1016/j.bpsc.2017.12.004. 29884281 PMC6054486

[brb371642-bib-0018] Kim, J. H. 2011. Resilience (Korean Resilience Quotient‐53). Wisdom House.

[brb371642-bib-0019] Kim, M. , S. Yun , H. Kim , et al. 2026. “Heart Rate Circadian Phase and Hyperarousal as Wearable Digital Phenotyping of Insomnia: An Interpretable Machine Learning Study.” Digital Health 12: 20552076261458929. 10.1177/20552076261458929. 42261286 PMC13242605

[brb371642-bib-0020] Kroenke, K. , R. L. Spitzer , and J. B. Williams . 2001. “The PHQ‐9: Validity of a Brief Depression Severity Measure.” Journal of General Internal Medicine 16, no. 9: 606–613. 10.1046/j.1525-1497.2001.016009606.x. 11556941 PMC1495268

[brb371642-bib-0021] Kroese, F. M. , D. T. De Ridder , C. Evers , and M. A. Adriaanse . 2014. “Bedtime Procrastination: Introducing a New Area of Procrastination.” Frontiers in Psychology 5: 611. 10.3389/fpsyg.2014.00611. 24994989 PMC4062817

[brb371642-bib-0022] Lee, Y. , S. Kim , S. Kim , S. P. Pack , H. J. Lee , and C. H. Cho . 2025. “Deep Phenotyping of Insomnia: A Multimodal Assessment Protocol.” Frontiers in Psychiatry 16: 1696593. 10.3389/fpsyt.2025.1696593. 41323485 PMC12661853

[brb371642-bib-0023] Matsushita, E. , H. Kim , M. G. Kim , S. J. Yoon , S. Kim , and J. W. Yeom . 2026. “Multidimensional Analysis of Smartphone Overuse in Insomnia: Integrating Digital Phenotyping With Clinical Assessment.” Journal of Behavioral Addictions 15, no. 1: 289–304. 41493477 10.1556/2006.2025.00093 PMC13132408

[brb371642-bib-0024] Morin, C. M. , and D. J. Buysse . 2024. “Management of Insomnia.” New England Journal of Medicine 391, no. 3: 247–258. 10.1056/NEJMcp2305655. 39018534

[brb371642-bib-0025] Morin, C. M. , and D. C. Jarrin . 2022. “Epidemiology of Insomnia: Prevalence, Course, Risk Factors, and Public Health Burden.” Sleep Medicine Clinics 17, no. 2: 173–191. 10.1016/j.jsmc.2022.03.003. 35659072

[brb371642-bib-0026] Morin, C. M. , A. Vallières , and H. Ivers . 2007. “Dysfunctional Beliefs and Attitudes About Sleep (DBAS): Validation of a Brief Version (DBAS‐16).” Sleep 30, no. 11: 1547–1554. 10.1093/sleep/30.11.1547. 18041487 PMC2082102

[brb371642-bib-0027] Rosenthal, N. E. , D. A. Sack , J. C. Gillin , et al. 1984. “Seasonal Affective Disorder: A Description of the Syndrome and Preliminary Findings With Light Therapy.” Archives of General Psychiatry 41, no. 1: 72. 10.1001/archpsyc.1984.01790120076010. 6581756

[brb371642-bib-0028] Roth, T. 2007. “Insomnia: Definition, Prevalence, Etiology, and Consequences.” Journal of Clinical Sleep Medicine 3, no. S5: S7–S10. 10.5664/jcsm.26929. 17824495 PMC1978319

[brb371642-bib-0029] Schwartz, J. E. , L. Jandorf , and L. B. Krupp . 1993. “The Measurement of Fatigue: A New Instrument.” Journal of Psychosomatic Research 37, no. 7: 753–762. 10.1016/0022-3999(93)90104-N. 8229906

[brb371642-bib-0030] Smith, C. S. , C. Reilly , and K. Midkiff . 1989. “Evaluation of Three Circadian Rhythm Questionnaires With Suggestions for an Improved Measure of Morningness.” Journal of Applied Psychology 74, no. 5: 728–738. 10.1037/0021-9010.74.5.728. 2793773

[brb371642-bib-0031] Spitzer, R. L. , K. Kroenke , J. B. W. Williams , and B. Löwe . 2006. “A Brief Measure for Assessing Generalized Anxiety Disorder.” Archives of Internal Medicine 166, no. 10: 1092. 10.1001/archinte.166.10.1092. 16717171

[brb371642-bib-0032] Yuh, C. , I.n‐S. Song , H.‐J. Lee , Y.‐M. Lee , and C.‐H. Cho . 2026. “Network Analysis of Sleep, Mental Health, and Stress in Korean Dentists.” International Dental Journal 76, no. 4: 109705. 10.1016/j.identj.2026.109705. 42341599 PMC13319880

