# Supplementary material for: Bipolarity as an Independent Correlate of Clinical Profiles in Insomnia: A Factorial Analysis Across Multiple Clinical Domains
Source: Brain Behav. 2026 Jul 28;16(8):e71642. doi: 10.1002/brb3.71642 (PMC13411293; doi:10.1002/brb3.71642)
Supplement: Supplementary file 1 — Supplementary Tables: brb371642‐sup‐0001‐TableS1‐S2.docx [file BRB3-16-e71642-s001.docx]

# **Supplementary Materials**

**Bipolarity as an independent correlate of clinical profiles in insomnia:** **a factorial analysis across multiple clinical domains**

Sujin Kim^a^, Seung Pil Pack^b^, Heon-Jeong Lee^a c d^, Chul-Hyun Cho^a c d^*****

^a^Department of Psychiatry, Korea University College of Medicine, 73 Goryeodae-ro, Seongbuk-gu, Seoul, Republic of Korea

^b^Department of Biotechnology and Bioinformatics, Korea University, 2511 Sejong-Ro, Sejong, Republic of Korea

^c^Department of Biomedical Informatics, Korea University College of Medicine, 161 Jeongneung-ro, Seongbuk-gu, Seoul, Republic of Korea

^d^Department of Medical Education, Korea University College of Medicine, 73 Goryeodae-ro, Seongbuk-gu, Seoul, Republic of Korea

***Corresponding Author:**

Chul-Hyun Cho, M.D., Ph.D.

Department of Psychiatry, Biomedical Informatics, and Medical Education, Korea University College of Medicine, Seoul, Republic of Korea

Address: 73, Goryeodae-ro, Seongbuk-gu, Seoul 02841, Korea

E-mail: david0203@korea.ac.kr; [david0203@gmail.com](mailto:david0203@gmail.com)

**Supplementary Table S1.** Two-way ANCOVA results with age, sex, and BMI as covariates

|  | **Sleep Status** | | | **Bipolarity** | | | **Interaction** | | |
| --- | --- | --- | --- | --- | --- | --- | --- | --- | --- |
| **Scale** | **F** | **pFDR** | **η²p** | **F** | **pFDR** | **η²p** | **F** | **pFDR** | **η²p** |
| PSQI | 96.75 | **<.001** | .230 | 2.55 | .141 | .008 | 1.87 | .852 | .006 |
| ESS | 5.79 | **.019** | .018 | 7.47 | **.018** | .023 | 0.43 | .882 | .001 |
| DBAS-16 | 31.62 | **<.001** | .089 | 6.88 | **.018** | .021 | 0.33 | .882 | .001 |
| BPS | 27.29 | **<.001** | .078 | 4.44 | **.056** | .014 | 0.10 | .882 | .000 |
| FIRST | 45.07 | **<.001** | .122 | 4.51 | .056 | .014 | 0.02 | .898 | .000 |
| PHQ-9 | 20.75 | **<.001** | .060 | 11.91 | **.003** | .035 | 1.42 | .852 | .004 |
| GAD-7 | 11.85 | **.001** | .035 | 15.83 | **.001** | .047 | 0.11 | .882 | .000 |
| BSQ | 4.22 | **.044** | .013 | 14.48 | **.001** | .043 | 0.42 | .882 | .001 |
| KRQ-53 | 9.16 | **.004** | .028 | 1.02 | .364 | .003 | 0.10 | .882 | .000 |
| CSM | 1.19 | .276 | .004 | 0.16 | .685 | .001 | 0.03 | .898 | .000 |
| K-BRIAN | 88.69 | **<.001** | .215 | 7.24 | **.018** | .022 | 0.30 | .882 | .001 |
| MFS | 38.35 | **<.001** | .106 | 3.65 | .080 | .011 | 3.98 | .656 | .012 |
| AUDIT-K | 28.91 | **<.001** | .082 | 0.67 | .447 | .002 | 0.10 | .882 | .000 |
| SPAQ GSS | 7.76 | **.007** | .023 | 11.29 | **.003** | .034 | 1.37 | .852 | .004 |

*Note. Covariates: age, sex, and BMI. Bold pFDR values indicate FDR-corrected p < .05. η²p = partial eta-squared. For scale abbreviations, see Table 2. Results are consistent with the primary analysis (Table 2), confirming that BMI does not materially alter the pattern of findings. FIRST and MFS bipolarity effects become marginal (pFDR = .056 and .080, respectively), but all other significant effects are preserved.*

**Supplementary Table S2.** Sensitivity analyses: Two-way ANCOVA results using alternative bipolarity cutoffs

**Panel A: K-MDQ ≥ 8 cutoff (n_High = 99, 29.3%)**

|  | **Sleep Status** | | | **Bipolarity** | | |
| --- | --- | --- | --- | --- | --- | --- |
| **Scale** | **F** | **pFDR** | **η²p** | **F** | **pFDR** | **η²p** |
| PSQI | 96.73 | **<.001** | .226 | 3.14 | .098 | .009 |
| ESS | 5.07 | **.027** | .015 | 9.86 | **.009** | .029 |
| DBAS-16 | 27.79 | **<.001** | .077 | 6.04 | **.034** | .018 |
| BPS | 26.53 | **<.001** | .074 | 2.32 | .150 | .007 |
| FIRST | 44.62 | **<.001** | .119 | 5.15 | **.039** | .015 |
| PHQ-9 | 21.25 | **<.001** | .060 | 8.99 | **.010** | .026 |
| GAD-7 | 10.43 | **.002** | .031 | 18.33 | **<.001** | .052 |
| BSQ | 5.07 | **.027** | .015 | 12.37 | **.003** | .036 |
| KRQ-53 | 9.83 | **.003** | .029 | 3.21 | .098 | .010 |
| CSM | 0.57 | .451 | .002 | 0.99 | .346 | .003 |
| K-BRIAN | 86.66 | **<.001** | .207 | 6.36 | **.034** | .019 |
| MFS | 35.58 | **<.001** | .097 | 5.70 | **.035** | .017 |
| AUDIT-K | 30.24 | **<.001** | .084 | 0.42 | .515 | .001 |
| SPAQ GSS | 6.47 | **.015** | .019 | 5.07 | **.039** | .015 |

**Panel B: K-MDQ ≥ 9 cutoff (n_High = 73, 21.6%)**

|  | **Sleep Status** | | | **Bipolarity** | | |
| --- | --- | --- | --- | --- | --- | --- |
| **Scale** | **F** | **pFDR** | **η²p** | **F** | **pFDR** | **η²p** |
| PSQI | 98.91 | **<.001** | .230 | 9.05 | **.008** | .027 |
| ESS | 5.86 | **.017** | .017 | 11.34 | **.005** | .033 |
| DBAS-16 | 29.37 | **<.001** | .081 | 7.95 | **.010** | .023 |
| BPS | 28.60 | **<.001** | .080 | 1.01 | .369 | .003 |
| FIRST | 45.94 | **<.001** | .122 | 10.94 | **.005** | .032 |
| PHQ-9 | 23.23 | **<.001** | .066 | 8.98 | **.008** | .026 |
| GAD-7 | 12.34 | **<.001** | .036 | 17.33 | **<.001** | .050 |
| BSQ | 6.42 | **.015** | .019 | 8.76 | **.008** | .026 |
| KRQ-53 | 9.02 | **.004** | .027 | 1.94 | .209 | .006 |
| CSM | 0.88 | .349 | .003 | 0.02 | .917 | .000 |
| K-BRIAN | 91.53 | **<.001** | .217 | 4.99 | **.041** | .015 |
| MFS | 38.38 | **<.001** | .104 | 3.71 | .077 | .011 |
| AUDIT-K | 32.08 | **<.001** | .088 | 0.01 | .917 | .000 |
| SPAQ GSS | 5.97 | **.017** | .018 | 5.29 | **.039** | .016 |

*Note. Covariates: age, sex. Bold pFDR values indicate FDR-corrected p < .05. Using ≥ 8 cutoff, 9 of 14 scales showed significant bipolarity effects (vs. 10 with ≥ 7 cutoff; BPS lost significance). Using ≥ 9 cutoff, 9 of 14 scales remained significant (BPS and MFS lost significance). The core findings—significant bipolarity effects on anxiety (GAD-7), anxiety sensitivity (BSQ), depression (PHQ-9), and seasonality (SPAQ GSS)—were robust across all cutoffs.*
